# *In Vitro*-Generated Tc17 Cells Present a Memory Phenotype and Serve As a Reservoir of Tc1 Cells *In Vivo*

**DOI:** 10.3389/fimmu.2018.00209

**Published:** 2018-02-08

**Authors:** Felipe Flores-Santibáñez, Bárbara Cuadra, Dominique Fernández, Mariana V. Rosemblatt, Sarah Núñez, Pablo Cruz, Felipe Gálvez-Cancino, J. César Cárdenas, Alvaro Lladser, Mario Rosemblatt, María Rosa Bono, Daniela Sauma

**Affiliations:** ^1^Departamento de Biologia, Facultad de Ciencias, Universidad de Chile, Santiago, Chile; ^2^Facultad de Medicina, Universidad San Sebastian, Santiago, Chile; ^3^Programa de Doctorado en Ciencias Biomedicas, Facultad de Medicina, Universidad de Chile, Santiago, Chile; ^4^Anatomy and Developmental Biology, Institute of Biomedical Sciences, University of Chile, Santiago, Chile; ^5^Fundacion Ciencia & Vida, Santiago, Chile; ^6^Geroscience Center for Brain Health and Metabolism, Santiago, Chile; ^7^Buck Institute for Research on Aging, Novato, CA, United States; ^8^Department of Chemistry and Biochemistry, University of California, Santa Barbara, Santa Barbara, CA, United States; ^9^Facultad de Ciencias Biologicas, Universidad Andrés Bello, Santiago, Chile

**Keywords:** Tc17 cells, CD8^+^ T cell memory, secondary expansion, persistence, homing, oxidative metabolism

## Abstract

Memory CD8^+^ T cells are ideal candidates for cancer immunotherapy because they can mediate long-term protection against tumors. However, the therapeutic potential of different *in vitro*-generated CD8^+^ T cell effector subsets to persist and become memory cells has not been fully characterized. Type 1 CD8^+^ T (Tc1) cells produce interferon-γ and are endowed with high cytotoxic capacity, whereas IL-17-producing CD8^+^ T (Tc17) cells are less cytotoxic but display enhanced self-renewal capacity. We sought to evaluate the functional properties of *in vitro*-generated Tc17 cells and elucidate their potential to become long lasting memory cells. Our results show that *in vitro*-generated Tc17 cells display a greater *in vivo* persistence and expansion in response to secondary antigen stimulation compared to Tc1 cells. When transferred into recipient mice, Tc17 cells persist in secondary lymphoid organs, present a recirculation behavior consistent with central memory T cells, and can shift to a Tc1 phenotype. Accordingly, Tc17 cells are endowed with a higher mitochondrial spare respiratory capacity than Tc1 cells and express higher levels of memory-related molecules than Tc1 cells. Together, these results demonstrate that *in vitro*-generated Tc17 cells acquire a central memory program and provide a lasting reservoir of Tc1 cells *in vivo*, thus supporting the use of Tc17 lymphocytes in the design of novel and more effective therapies.

## Introduction

Effector CD8^+^ T cells can be classified into at least two functional subsets. Type 1 CD8^+^ T (Tc1) cells produce interferon (IFN)-γ and are endowed with high cytotoxic capacity. Type 17 CD8^+^ T (Tc17) cells produce IL-17 and display enhanced self-renewal potential. The presence of Tc17 cells has been described in several infectious and autoimmune diseases in both human and mice (1, 2), thus confirming the role of Tc17 cells in inflammatory processes. The accumulation of Tc17 lymphocytes has also been observed in several types of human tumors including gastric (3), uterus (4), head and neck (5), liver (6), and colon cancer (7) and also in various cancer models such as B16 melanoma (8). In spite of their relatively low abundance in several tumors, we and others have reported that the adoptive transfer of *in vitro*-generated Tc17 cells promotes the elimination of established tumors in C57BL/6 mice, supporting evidence of antitumor properties of Tc17 lymphocytes (9–12).

The fact that *in vitro*-generated Tc17 lymphocytes do not express cytotoxic molecules such as granzymes and perforins (13) and thus are not able to induce apoptosis of target cells (14) opens questions regarding the mechanisms that could explain the antitumor potential of Tc17 lymphocytes *in vivo*. In search of evidence which may account for this dichotomy, we demonstrated that Tc17 cells are highly plastic and some cells differentiate into Tc1 IFN-γ^+^/granzyme B^+^ cells upon transfer into tumor-bearing mice (12). Moreover, Restifo and collaborators further suggested that the effectiveness of Tc17 lymphocyte adoptive therapy depends on the ability of these cells to differentiate into IFN-γ-producing cells (9). Accordingly, Dutton’s group reported that after adoptive transfer, Tc17 cells promote the recruitment of macrophages and neutrophils to the tumor microenvironment through the production of IFN-γ, TNF-α, and IL-17 (10).

Recently, it has been suggested that Tc17 cells may display phenotypic features of memory T cells. The group of Takiguchi described that human Tc17 cells are found within the CD28^+^ CD45RA^−^ memory subset in healthy donors (15). Also, other reports have shown that both endogenous and *in vitro*-generated Tc17 cells express high levels of the TCF-7 transcription factor (16, 17), suggesting an active Wnt/β-catenin signaling, which constitutes a major pathway in maintaining the molecular program of memory T lymphocytes. In the context of cancer, it has been reported that adoptively transferred Tc17 lymphocytes have greater persistence *in vivo* compared to Tc1 lymphocytes (9) and that Tc17 lymphocytes exhibit high IL-7 receptor alpha chain (CD127) expression (11), which may explain their increased survival capacity. Finally, it has been shown that Tc17 cells can differentiate into tissue-resident memory T cells after the resolution of psoriasis (18). However, it is unclear whether Tc17 lymphocytes possess other functional features of memory cells, such as the ability to mount a secondary response and an OXPHOS-based metabolism.

Based on these previous findings, we sought to evaluate whether Tc17 cells present functional properties of memory CD8^+^ T cells. For this, we generated OVA-specific Tc17 cells and transferred the cells into wild-type mice to test their capacity to expand upon a secondary challenge. We show that Tc17 cells persist in the long term, expand upon a secondary stimulation with OVA, shift to a Tc1 phenotype and recirculate within secondary lymphoid organs. In agreement, Tc17 cells are endowed with a higher mitochondrial spare respiratory capacity (SRC) compared to conventional (Tc1) cytotoxic CD8^+^ T cells. Finally, Tc17 cells express higher levels of memory-related markers and lower levels of molecules required for the effector program compared to Tc1 cells. Together, these results suggest that Tc17 cells present functional properties of central memory CD8^+^ T cells.

## Materials and Methods

### Mice

C57BL/6 (CD45.2^+^), B6SJL-PTPRC (CD45.1^+^), OT-I, and Rag1^−/−^ mice were purchased from The Jackson Laboratory. All mice were kept in the animal facility at Fundación Ciencia y Vida and maintained according to the “Guide to Care and Use of Experimental Animals, Canadian Council on Animal Care.” Animal work was carried out under institutional regulations of Fundacion Ciencia & Vida and Facultad de Ciencias, Universidad de Chile and was approved by the local ethics review committees.

### Generation of Tc17 and Tc1 Cells

Naive CD8^+^ T cells were purified from spleens and lymph nodes of OT-I mice. Briefly, the spleen was perfused with RPMI 1640 supplemented with 10% FCS, and CD8^+^ T cells were negatively selected using MACS (Miltenyi Biotec) following the manufacturer’s instructions. Following enrichment of CD8^+^ T cells, naive CD8^+^ T cells (CD8^+^/CD44lo/CD62Lhi/CD25^−^) were obtained by cell sorting (FACS Aria III, BDBiosciences). Naive CD8^+^ T cells were cultured in a 96-well round bottom microplate (10^5^ CD8^+^ T cells/well) and were activated with soluble α-CD3 (1 µg/ml; clone 145-2C11, eBioscience) and α-CD28 (1 µg/ml; clone 37.51) for 4 days in the presence of different cytokine cocktails. To generate Tc17 cells, CD8^+^ T cells were differentiated for 4 days in the presence of 2 ng/ml recombinant human TGF-β1 (eBioscience), 20 ng/ml recombinant mouse IL-6 (eBioscience), and 5 µg/ml of anti-IFN-γ (clone XMG1.2, Biolegend). Tc1 cells were differentiated for 4 days in the presence of 10 ng/ml recombinant mouse IL-2 (eBioscience) using a protocol modified from the literature which replicates the clinical protocol of expanding human tumor infiltrating lymphocytes (13). Cells were then harvested for *in vitro* experiments, adoptive transfer experiments, RNA extraction, intracellular cytokine staining, and flow cytometry.

### Intracellular Staining and Flow Cytometry

To assess cytokine production by adoptively transferred Tc1 and Tc17 cells, cells obtained from lymph nodes, spleen, and peritoneal cavity of recepient mice were re-stimulated with 0.25 µM PMA (Sigma-Aldrich) and 1 µg/ml ionomycin (Sigma-Aldrich) in the presence of GolgiPlug (BD Biosciences) for 4 h. Cells were stained with antibodies against the cell surface markers (congenic markers) and then resuspended in a fixation/permeabilization solution (Cytofix/Cytoperm; BD Pharmingen). Following fixation and permeabilization, the cells were incubated with antibodies against IFN-γ, IL-2, IL-17, and TNF-α for 30 min at 4°C. The cells were then washed with permeabilization buffer and resuspended in PBS supplemented with 2% FCS for FACS analysis (FACSCanto II; BD Bioscience). In some cases, Fixable Viability Dye (eBioscience) was used to discard dead cells from the analysis.

Intracellular staining for Ki67 was conducted by incubating surface stained cells in a fixation/permeabilization solution (Foxp3/transcription factor permeabilization and fixation buffer, eBioscience). Following fixation and permeabilization, the cells were incubated with the Ki-67 antibody (clone 11F6, Biolegend) in a permeabilization buffer (Foxp3/transcription factor permeabilization buffer, eBioscience). After staining, cells were washed and analyzed by FACS. FACS data analysis was performed using the FLOWJO software (Tree Star Inc., Ashland, OR, USA).

### qPCR

Tc1 and Tc17 cells were differentiated *in vitro*, and total RNA was extracted. Naive CD8^+^ T cells were isolated by cell sorting (CD8^+^/CD44lo/CD62Lhi/CD25^−^) before RNA extraction. Total RNA was obtained using EZNA Total RNA Kit I (Ω Bio-Tek). RNA (1 µg) was reverse-transcribed using M-MLV reverse transcriptase (Promega). The PCR reaction was performed using Brilliant II SYBR Green QPCR Master Mix (Agilent Technologies) in a Stratagene Mx3000P real-time PCR machine. For relative quantitation, the amplified fragments were normalized according to constitutive transcription of the housekeeping gene HPRT. The sequences of the primers used for quantification of each measured transcript were the following:

tcf7 forward 5′- CAA TCT GCT CAT GCC CTA CC -3′, reverse 5′- CTT GCT TCT GGC TGA TGT CC -3′; lef1 forward 5′- TGA GTG CAC GCT AAA GGA GA -3′, reverse 5′- CTG ACC AGC CTG GAT AAA GC -3′; bcl6 forward 5′- CTG CAG ATG GAG CAT GTT GT -3′, reverse 5′- CAC CCG GGA GTA TTT CTC AG -3′; klf2 forward 5′- TGT GAG AAA TGC CTT TGA GTT TAC TG -3′, reverse 5′- CCC TTA TAG AAA TAC AAT CGG TCA TAG TC-3′; tbx21 forward 5′- CCT GTT GTG GTC CAA GTT CA AC-3′, reverse 5′- CAC AAA CAT CCT GTA ATG GCT TGT -3′; eomes forward 5′- GCG CAT GTT TCC TTT CTT GAG -3′, reverse 5′- GGT CGG CCA GAA CCA CTT C -3′; prdm1 forward 5′- GAC GGG GGT ACT TCT GTT CA -3′, reverse 5′- GGC ATT CTT GGG AAC TGT GT -3′; grzb forward 5′- ATC AAG GAT CAG CAG CCT GA-3′, reverse 5′- TGA TGT CAT TGG AGA ATG TCT -3′; pfn1 forward 5′- GAT GTG AAC CCT AGG CCA GA -3′, reverse 5′- TGA TGT CAT TGG AGA ATG TCT -3′; hprt forward 5′- CTC CTC AGA CCG CTT TTT GC -3′, reverse 5′- TAA CCT GGT TCA TCA TCG CTA ATC -3′.

### Western Blot

Cell lysis was performed using Cytobuster protein extraction reagent (Novagen) supplemented with protease and phosphatase inhibitors (complete PhosSTOP, Roche). Protein extracts were separated in 15% SDS-polyacrylamide gels and transferred to PDVF membranes (Millipore). Blocking was at room temperature for 1 h in 5% fat-free milk, and membranes were incubated overnight at 4°C with 1:2,000 dilution of Total OXPHOS Human WB Antibody Cocktail (ab110411), and then for 1 h at room temperature with a secondary antibody conjugated to horseradish peroxidase. Chemiluminescence detection used SuperSignal West Pico Chemiluminescent Substrate (Thermo Scientific) and series of timed exposure images were acquired with a Chemidocs imaging system (BioRad) to ensure densitometric analyses were performed at exposures within the linear range. To ensure equal protein loading across gels, membranes were submerged in stripping buffer (Restore Western blot stripping buffer; Pierce), incubated at 37°C for 30 min, and re-probed with a loading control antibody. Image J was used for densitometric analysis.

### Adoptive Transfer of Tc1 and Tc17 Cells

To assess secondary expansion of transferred cells, Tc1 and Tc17 lymphocytes were generated *in vitro* from OT-I/CD45.2^+^ and OT-I CD45.1^+^/CD45.2^+^ mice, respectively. On the day of the transfer, the Tc1 and Tc17 lymphocytes were counted and mixed at a 1:1 ratio. The initial ratio (input) on the cytometer was then evaluated by staining against CD45.1 and CD45.2. A total of 2 × 10^6^ cells from this mixture were transferred intravenously into B6.SJL (CD45.1^+^) congenic mice. At day 23 post-adoptive transfer, the mice were immunized with 500 µg of OVA protein (Sigma-Aldrich) and 100 µl of Freund’s Complete Adjuvant (CFA, Sigma) intraperitoneally. On days 10, 20, and 27 post-adoptive transfer blood samples were obtained and analyzed by flow cytometry. Finally, on day 27 after the adoptive transfer, the mice were sacrificed, and cells obtained from the peritoneal cavity, secondary lymphoid organs, and spleen were analyzed by FACS.

To examine the ability of Tc17 cells to be retained in effector tissues, we performed intradermal injections of *in vitro*-differentiated CD45.2^+^ Tc1 and CD45.1^+^/CD45.2^+^ Tc17 cells (1.5 × 10^6^ cells per mice, at a 1:1 ratio) into CD45.1^+^ congenic mice, and 30 days later, we analyzed skin, secondary lymphoid organs, spleen, bone marrow, lung, and liver for the transferred cells. The intradermal transfer was performed by injecting 100 µl of cellular suspension in PBS divided in five spots of 20 µl each in the lower back skin of each mouse.

### Homeostatic Survival

For *in vitro* homeostatic survival analysis, Tc1 and Tc17 cells were cultured in the presence of IL-15 (25 ng/ml, eBioscience) or IL-7 (10 ng/ml, eBioscience). Three days later, cells were harvested, counted, and analyzed by FACS.

For *in vivo* homeostatic survival analysis, Tc1 and Tc17 lymphocytes were generated *in vitro* from CD45.2^+^ and CD45.1^+^/CD45.2^+^ mice, respectively, and co-transferred (0.5 × 10^6^ cells of each type) in Rag1^−/−^ mice. The transferred cells were evaluated 10, 25, 75, and 115 days after in blood samples using counting beads (eBioscience).

### Homing Assay

Tc1 and Tc17 lymphocytes were generated *in vitro* from CD45.2^+^ mice and stained with eFluor 670 (Thermofisher Scientific, 5 µM) and CMTMR (Thermofisher Scientific, 10 µM), respectively. Then, the cells were co-transferred (0.5 × 10^6^ cells of each type) in CD45.1^+^ mice. Homing was evaluated 24 h later. Homing index in each organ was calculated as (Tc17_organ_/Tc1_organ_)/(Tc17_input_/Tc1_input_).

### Seahorse

Oxygen consumption rates (OCRs) and extracellular acidification rates (ECARs) from *in vitro* generated Tc1 and Tc17 cells (0.5 × 10^6^ cells/well) were measured in non-buffered DMEM without phenol red (containing 25 mM glucose, 2 mM l-glutamine, and 1 mM sodium pyruvate) under basal conditions and in response to 1 µM oligomycin, 0.5 µM fluoro-carbonyl cyanide phenylhydrazone, and 1 µM rotenone + 1 µM antimycin A (Sigma). The cells were analyzed with the XFe-96 Extracellular Flux Analyzer (Seahorse Bioscience).

### Statistical Analysis

Data are presented as mean ± SEM. Differences between two groups were determined using two-tailed Mann–Whitney test. Differences between more than two groups were determined using one-way ANOVA with Tukey or Bonferroni post-test. A two-way ANOVA with Bonferroni post-test was used to compare secondary expansion rates. Statistical analysis and graphs were obtained with GRAPHPAD PRISM (GraphPad Software Inc).

## Results

### Tc17 Cells Expand Rapidly following a Secondary Challenge

**Figure 1:**
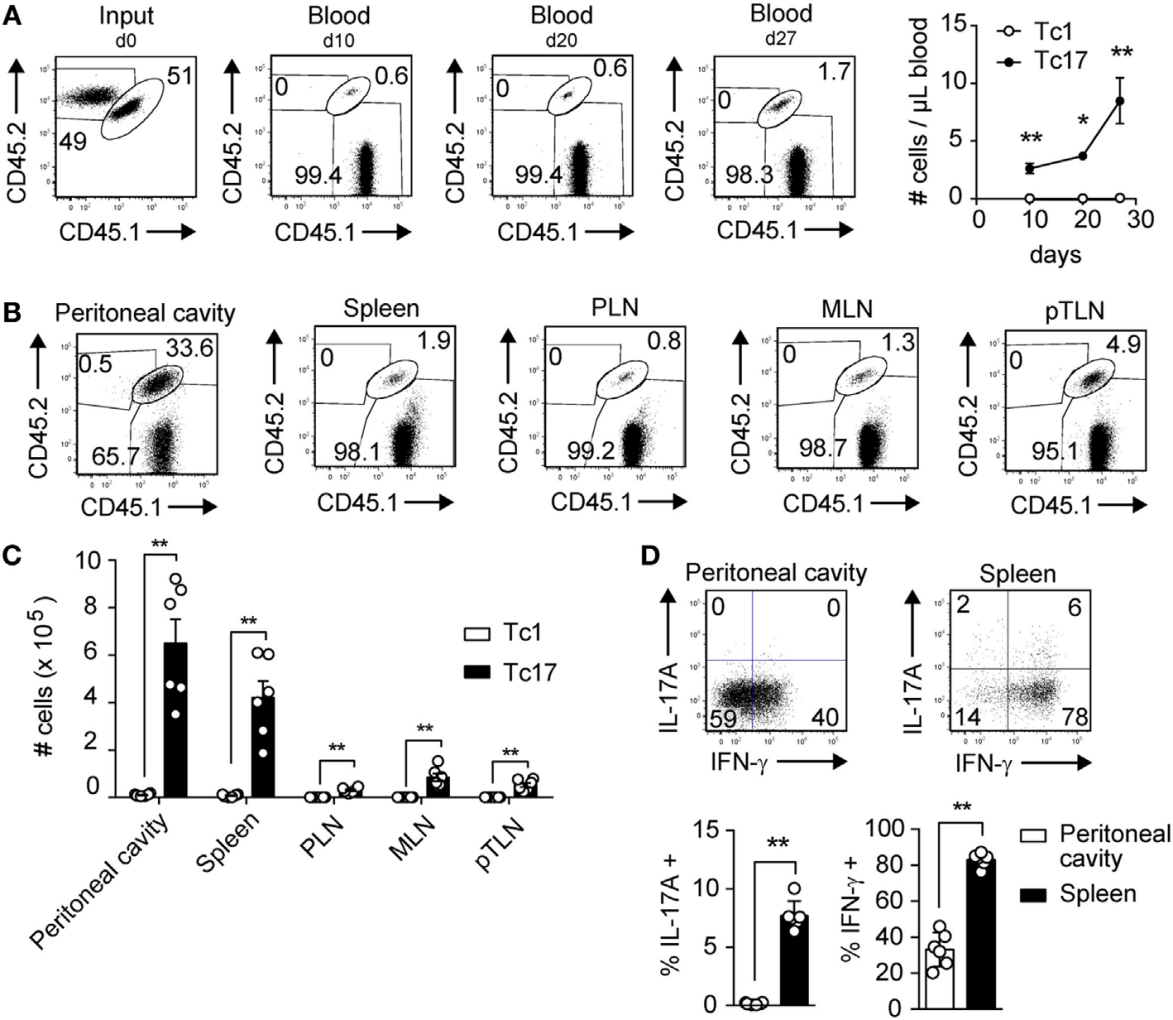
Tc17 cells persist and expand rapidly following a challenge *in vivo*. Tc1 cells (CD45.2^+^) and Tc17 cells (CD45.1^+^/CD45.2^+^) were generated *in vitro* from OT-I mice. The cells were co-injected (2 × 10^6^ total cells) into CD45.1^+^ recipient mice. The mice were immunized i.p. with OVA protein + CFA at day 23 following adoptive transfer of Tc1 and Tc17 cells. **(A)** Left, dot plot depicting the input and the frequencies of Tc1 and Tc17 cells in blood at days 10, 20, and 27 after adoptive transfer. Right, number of transferred Tc1 (white circles) and Tc17 cells (black circles) per microliter of blood at days 10, 20, and 27. Two-tailed Mann–Whitney test *p < 0.05, **p < 0.01, *n* = 4–5. **(B)** Dot plots depicting transferred Tc1 and Tc17 cells in peritoneal cavity, spleen, peripheral lymph nodes (PLN), mesenteric lymph node (MLN), and parathymic lymph nodes (pTLN) at day 27 after adoptive transfer. **(C)** Number of transferred Tc1 (white bars) and Tc17 cells (black bars) at day 27 in peritoneal cavity, spleen, PLN, MLN, and pTLN. Two-tailed Mann–Whitney test **p < 0.01, *n* = 6. **(D)** Cytokine production following PMA plus ionomycin stimulation was analyzed on transferred Tc17 cells (CD45.1^+^/CD45.2^+^ cells) obtained from spleen and peritoneal cavity of recipient mice. Two-tailed Mann–Whitney test **p < 0.01, *n* = 6.

In a previous study, we have demonstrated that Tc17 cells are endowed with high antitumor capacity and persistence, despite displaying a reduced cytotoxic potential *in vitro* (12). These observations lead us to hypothesize that Tc17 cells present a memory phenotype. To test this hypothesis, we co-transferred *in vitro*-generated Tc1 and Tc17 cells obtained from OT-I mice into wild type mice and then 3 weeks later, the mice were immunized i.p. with OVA-CFA. We analyzed the persistence and expansion of Tc1 and Tc17 cells before and after OVA immunization. As shown in Figure 1A, 10 and 20 days after the co-transfer, we could only detect the presence of Tc17 cells in blood, and these cells suffered a two-fold expansion following immunization with OVA, whereas Tc1 cells remained undetected. Accordingly, Tc17 cells were mainly found in the peritoneal cavity while Tc1 cells were present in low frequencies, ruling out the possibility that Tc1 cells were absent in blood because they were more efficiently recruited to the peritoneal cavity (Figures 1B,C). Interestingly, besides being detected in the peritoneal cavity, Tc17 cells were also present in spleen and secondary lymph nodes such as peripheral lymph nodes (PLN), mesenteric lymph node (MLN), and parathymic lymph nodes (pTLN) (Figures 1B,C). This observation suggests that Tc17 cells may be recruited to effector tissues but also, that a portion of Tc17 cells may remain circulating within secondary lymph nodes as a reservoir for future encounter with their cognate antigen. Next, we tested whether Tc17 cells continued to produce IL-17 or could give rise to IFN-γ-producing cells. As shown in Figure 1D, a high percentage of transferred Tc17 cells differentiated into IFN-γ-producing cells in spleen and peritoneal cavity, whereas a small fraction of Tc17 cells continued to produce IL-17 in spleen, confirming their high plasticity in this setting. Tc17 cells also expanded more rapidly and outnumbered Tc1 cells following *in vitro* polyclonal activation with anti-CD3 and anti-CD28 antibodies (data not shown). Taken together, these results demonstrate the ability of Tc17 cells to persist in the long term and rapidly expand upon a challenge.

Next, we evaluated whether Tc17 cells could differentiate to memory cells *in vivo* which could expand rapidly following a re-challenge with their cognate antigen. For this, we co-transferred *in vitro*-generated Tc1 and Tc17 cells obtained from OT-I mice into wild-type mice, and 24 h later, the mice were challenged with OVA. Four weeks later, mice were re-challenged with OVA and the frequencies of transferred cells in blood, PLN and spleen were analyzed. In this setting, the composition of Tc1 and Tc17 cells remained similar to the input before the re-challenge with OVA (dotted line, Figure S1C in Supplementary Material). However, following re-challenge with OVA protein (day 34), we found that Tc17 cells rapidly expanded (Figures S1A,B in Supplementary Material) and became almost 90% of transferred cells (Figure S1C in Supplementary Material), confirming that Tc17 cells are superior than Tc1 cells in proliferating in response to their cognate antigen. Similar results were obtained when analyzing the composition of transferred cells in spleen and PLN (Figure S1C in Supplementary Material). Interestingly, 1 week after the re-challenge, Tc17 cells found in the spleen and PLN gave rise to IFN-γ-producing cells (54.6 ± 7.3%) that do not express KLRG1 but express high levels of CD44 and CD127 (Figure S1D in Supplementary Material). Moreover, we found that a proportion of these Tc17 cells (29.6 ± 3.7%) express CD62L, which explains their potential to migrate within secondary lymph nodes (Figure S1D in Supplementary Material).

### Tc17 Cells Present a Migration Pattern of Central Memory T Cells

**Figure 2:**
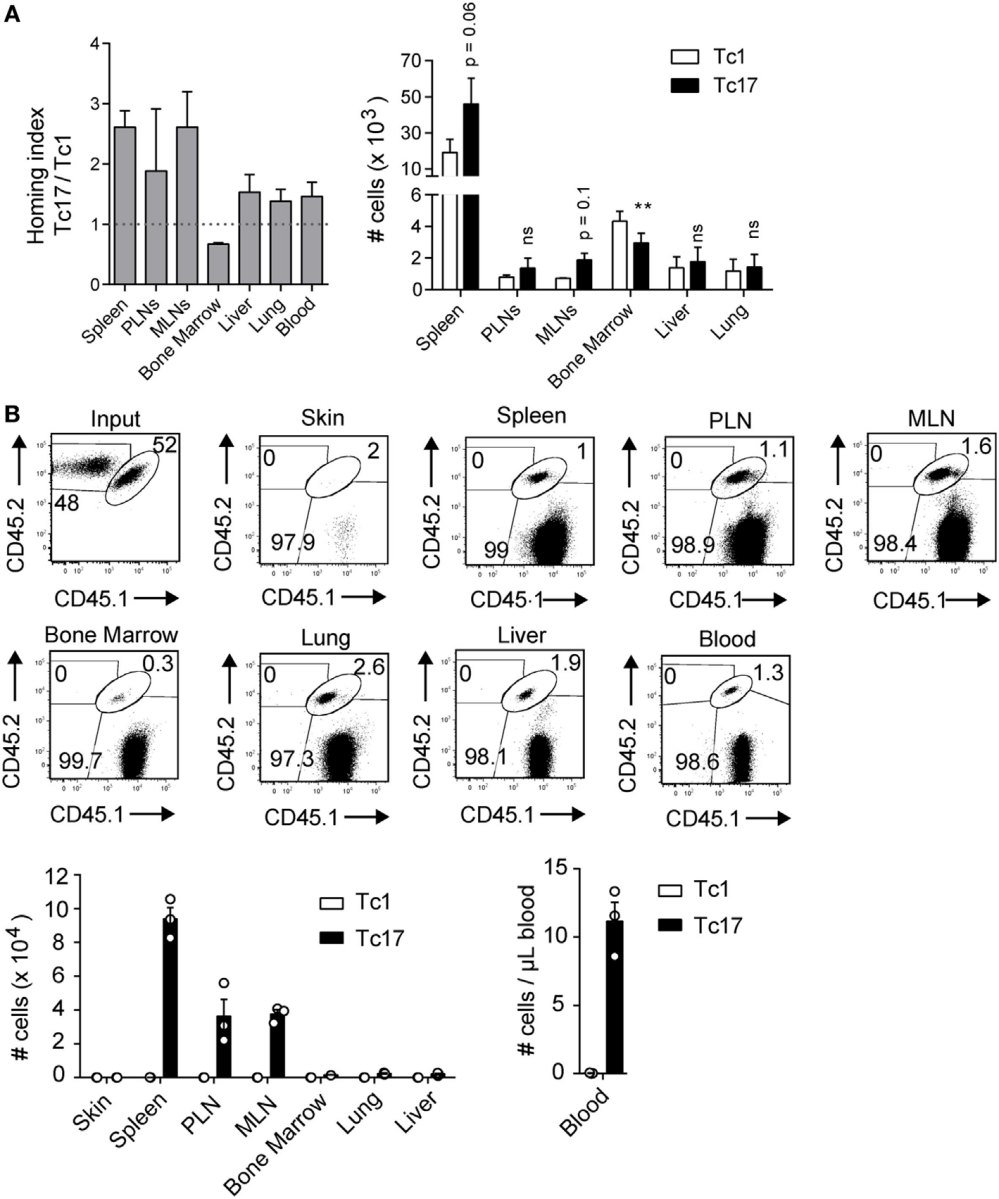
Tc17 cells recirculate within secondary lymph nodes. **(A)** Tc1 and Tc17 lymphocytes were generated *in vitro* from CD45.2^+^ mice and stained with eFluor 680 (5 µM) and CMTMR (10 µM) respectively. Then the cells were co-transferred (0.5 × 10^6^ cells of each type) in CD45.1^+^ mice. Cell migration was evaluated 24 h later in several lymphoid and effector tissues. Homing index in each organ was calculated as (Tc17_organ_/Tc1_organ_)/(Tc17_input_/Tc1_input_), *n* = 3. **(B)** Tc1 cells (CD45.2^+^) and Tc17 cells (CD45.1^+^/CD45.2^+^) were generated *in vitro* and injected intradermally into congenic mice (CD45.1^+^). 32 days later, the number of transferred cells was analyzed in skin, spleen, peripheral lymph nodes (PLN), mesenteric lymph node (MLN), bone marrow, lung, liver, and blood of recipient mice, *n* = 3.

In agreement with our previous observations in tumor bearing mice (12), we demonstrated that transferred Tc17 cells were always found in the spleen and secondary lymphoid organs (Figure 1B; Figure S1C in Supplementary Material), suggesting that they have a circulation pattern similar to central memory (T_CM_) cells. To study the migration pattern of Tc17 cells, *in vitro*-generated Tc17 and Tc1 cells were stained and transferred into congenic mice. 24 h later, we analyzed spleen, PLN, MLN, bone marrow, liver, lung, and blood for the presence of the transferred cells. As shown in Figure 2A, Tc17 cells migrated preferentially to secondary lymphoid organs such as the spleen, PLN, and MLN compared to Tc1 cells. In contrast, Tc1 cells preferentially migrated to the bone-marrow compared to Tc17 cells (Figure 2A).

Next, we tested whether Tc17 cells injected in peripheral tissues can become established as memory cells in secondary lymphoid organs. For this, we intradermally co-transferred Tc1 and Tc17 cells in a 1:1 ratio into CD45.1^+^ mice and 30 days later, the presence of memory cells was analyzed in skin, spleen, PLN, MLN, bone marrow, liver, lung, and blood. As shown in Figure 2B, we did not find Tc1 or Tc17 cells in the skin, however, Tc17 cells were found in blood and in all the organs analyzed. Interestingly, when analyzing the absolute numbers, we observed that Tc17 cells were preferentially found in secondary lymphoid organs (Figure 2B). These results demonstrate that Tc17 cells are not retained in the skin but migrate into secondary lymphoid organs.

### Tc17 Cells Proliferate More Robustly than Tc1 Cells in Response to Homeostatic Stimuli

**Figure 3:**
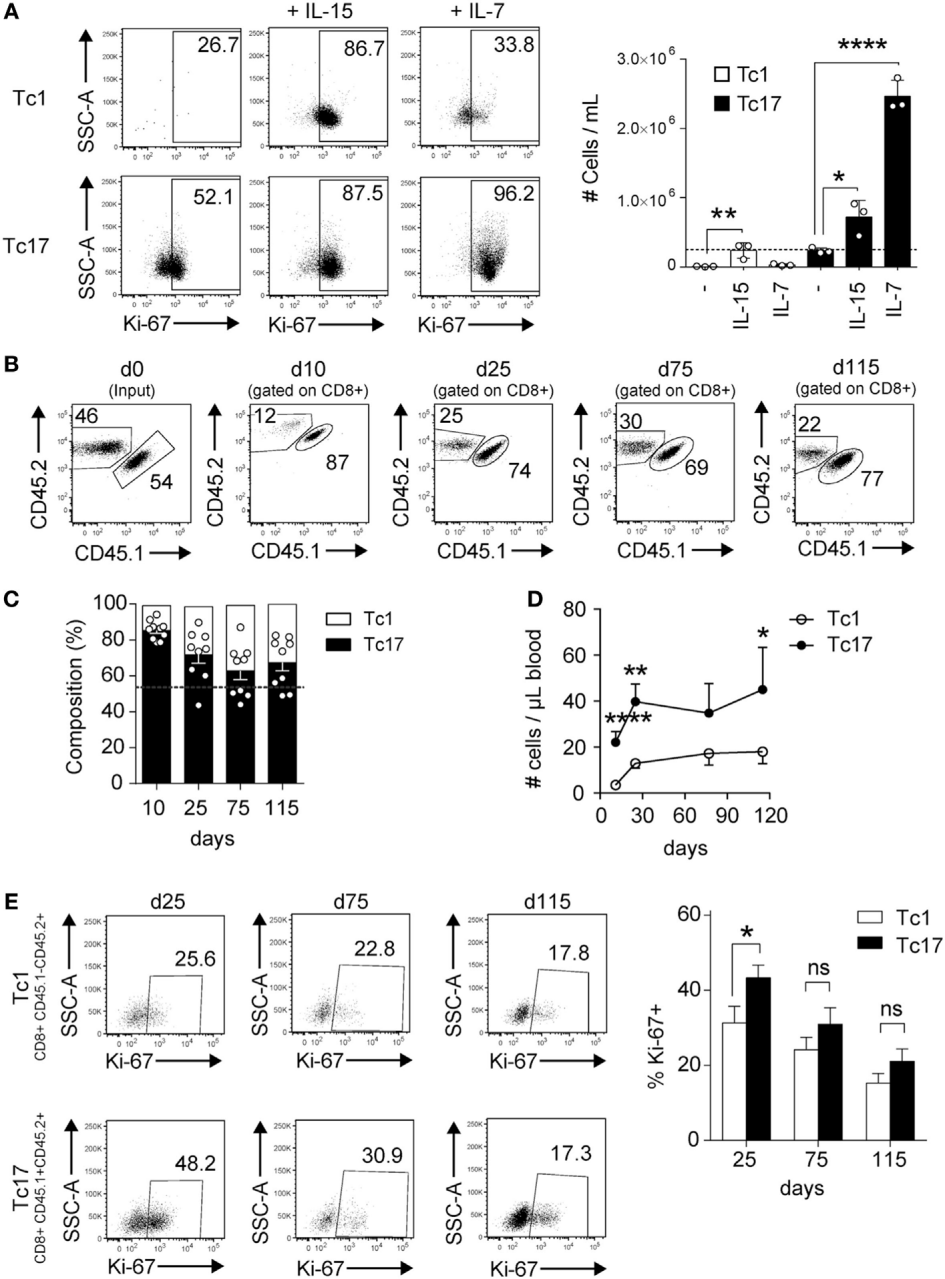
Tc17 cells proliferate in response to homeostatic stimuli. **(A)** *In vitro*-generated Tc1 and Tc17 cells were cultured in the presence of IL-15 (25 ng/ml) or IL-7 (10 ng/ml). 3 days later, cells were harvested and analyzed by FACS for Ki-67 staining. Graph on the right shows the number of cells per milliliter obtained following 3 days of culture. The dotted line represents the number of cells cultured at day 0. One-way ANOVA and Bonferroni post-test (control versus treated), *p < 0.05, ****p < 0.0001, *n* = 3. **(B–E)** Tc1 and Tc17 lymphocytes were generated *in vitro* from CD45.2^+^ and CD45.1^+^/CD45.2^+^ mice, respectively, and co-transferred (0.5 × 10^6^ cells of each type) into Rag1^−/−^ mice. **(B)** Dot plots show the percentage of transferred Tc1 and Tc17 cells in blood at different time points. **(C)** Composition of transferred Tc1 and Tc17 cells in blood at different time points. Dotted line represents the input. **(D)** Number of transferred Tc1 and Tc17 cells per microliter of blood. **(E)** Percentage of Ki-67^+^ cells within Tc1 and Tc17 cells in blood. Pooled data from two independent experiments (*n* = 9–10). Two-tailed Mann–Whitney test *p < 0.05, **p < 0.01, ****p < 0.0001.

To analyze the homeostatic response of Tc17 cells, we cultured Tc17 and Tc1 cells with the homeostatic cytokines IL-15 and IL-7, and the proliferation and absolute numbers of cells were analyzed 3 days later. As shown in Figure 3A, Tc17 cells responded to both homeostatic cytokines while Tc1 cells proliferated only in response to IL-15, which is in agreement with the fact that only Tc17 cells express CD127. In spite of their rapid proliferation, Tc17 cells retained the ability to produce IL-17 and only a small fraction of cells produced IFN-γ (data not shown).

To further analyze the *in vivo* homeostatic proliferation of Tc17 cells, Tc1 and Tc17 cells were transferred into Rag1^−/−^ mice at a 1:1 ratio and several days later, the absolute number of transferred cells was analyzed. As shown in Figures 3B–D, both Tc1 and Tc17 cells were present in Rag1^−/−^ mice 11 and 25 days following the transfer, but the absolute number of Tc17 cells in blood always doubled the absolute number of Tc1 cells. Moreover, a higher percentage of Tc17 cells were positive for Ki67 staining at day 25 compared to Tc1 cells (Figure 3E). Thus, Tc17 cells are much more responsive to homeostatic stimulation than Tc1 cells.

### Tc17 Cells Present a Transcriptional and Metabolic Profile of Memory T Cells

**Figure 4:**
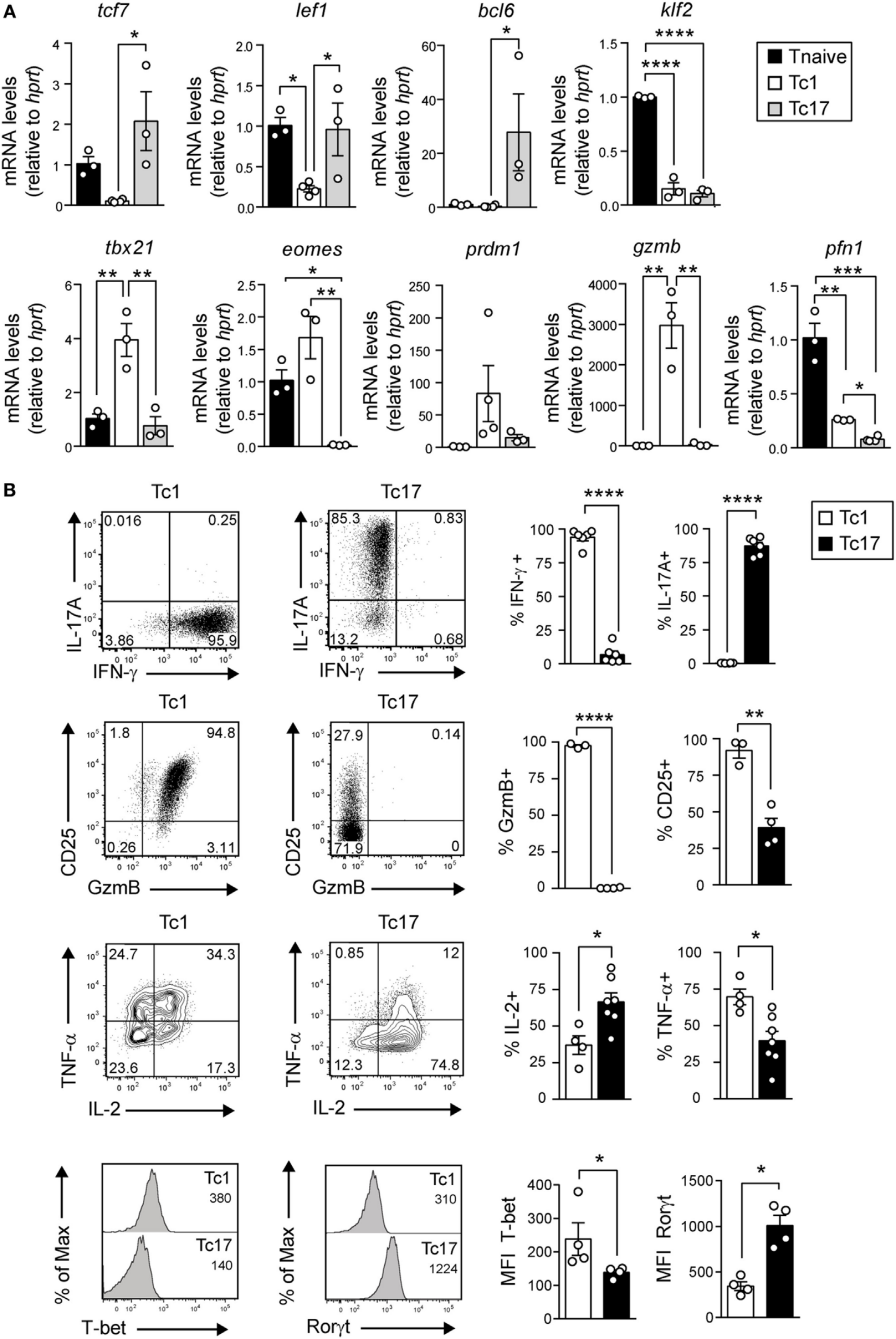
Tc17 cells present a memory-like phenotype. **(A)** The expression of memory and effector CD8^+^ T cell markers was analyzed by real-time PCR from cell-sorted naive CD8^+^ T cells and *in vitro*-generated Tc1 and Tc17 cells. The mRNA level was calculated relative to the expression of the housekeeping gene HPRT and normalized by the expression of naive T cells. One-way ANOVA and Tukey post-test, *p < 0.05, **p < 0.01, ***p < 0.001, ****p < 0.0001, *n* = 3–4. **(B)** Expression of IL-17A, interferon (IFN)-γ, CD25, GzmB, IL-2, TNF-α, T-bet, and Rorγt by *in vitro*-generated Tc1 and Tc17 cells measured by FACS. Two-tailed Mann–Whitney test *p < 0.05, **p < 0.01, ****p < 0.0001, *n* = 3–7.

We have previously shown that Tc17 cells express higher levels of tcf-7 and lef1 and lower levels of GzmB compared to Tc1 cells (12). To further characterize the phenotype of Tc17 cells, *in vitro*-generated Tc1 and Tc17 cells were analyzed by real-time PCR and by FACS in search of additional markers related to memory cells and effector cells. As shown in Figure 4A, we observed that Tc17 cells express markers related to memory T cells such as bcl-6 and lack markers of terminal differentiation such as tbx-21, eomes, and prdm1. On the other hand, Tc1 and Tc17 cells did not express Klf-2 transcriptional factor, which commands CD62L expression. Interestingly, in contrast to Tc1 cells, Tc17 cells present low levels of CD25, granzyme B, and TNF-α, while they express RORγt and produce high levels of IL-2 (Figure 4B).

**Figure 5:**
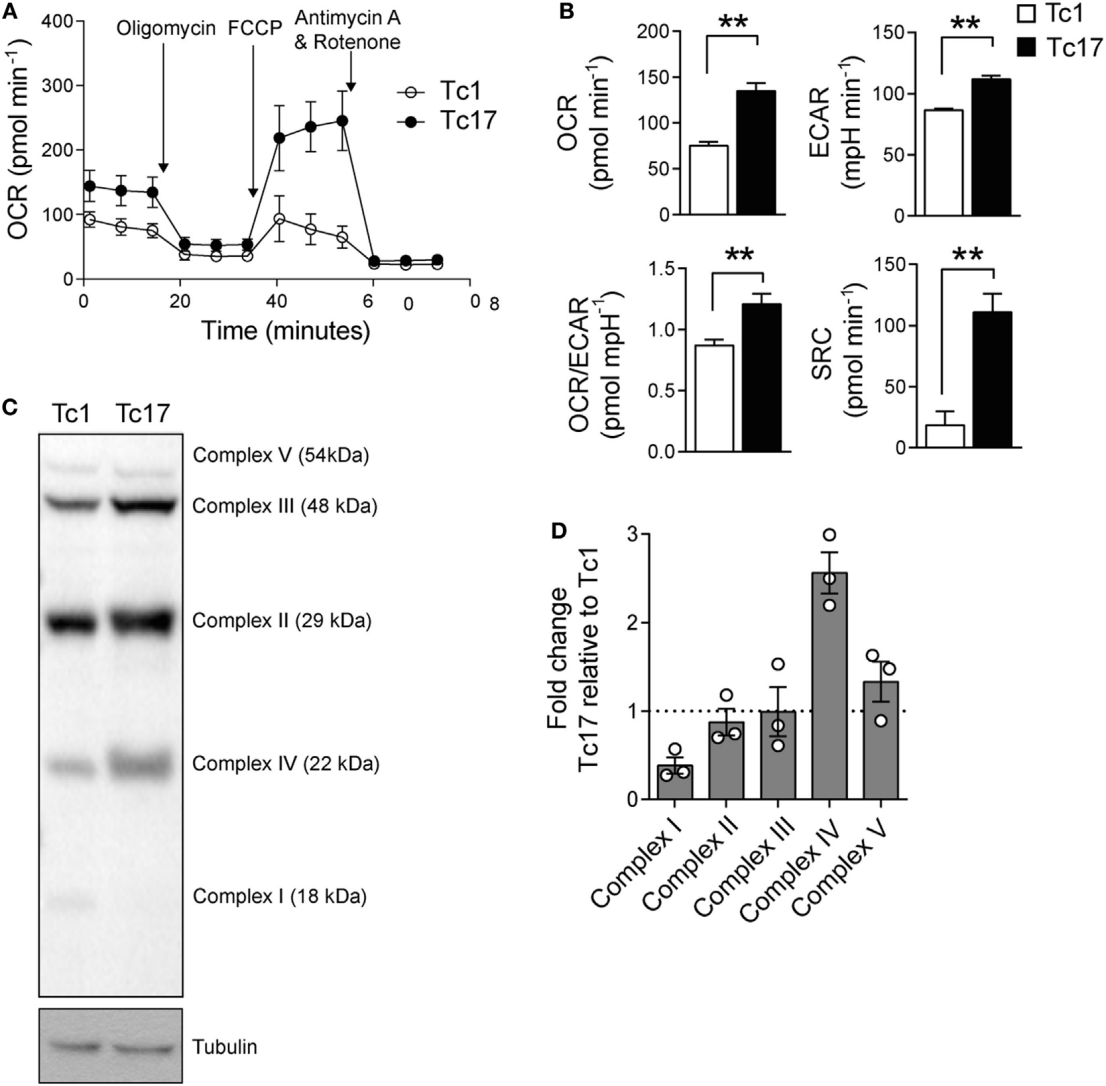
Tc17 cells present a metabolic profile of memory T cells. **(A,B)** Oxygen consumption rates (OCRs) and extracellular acidification rates (ECARs) from *in vitro*-generated Tc1 and Tc17 cells were measured under basal conditions and in response to 1 µM oligomycin, 0.5 µM fluoro-carbonyl cyanide phenylhydrazone, and 1 µM rotenone+ 1 µM antimycin A. Representative of two independent experiments. Two-tailed Mann–Whitney test, **p < 0.01, *n* = 6–7. **(C)** ETC Complex I, II, III, IV, and V proteins were analyzed by western blot in Tc17 and Tc1 cells. Representative of three independent experiments. **(D)** Quantification of ETC complexes, *n* = 3.

Since Tc17 cells express high levels of bcl-6 and low levels of prdm1 which suggests that Tc17 cells present a metabolic signature related to memory T cells, we sought to determine the metabolic profile of Tc17 cells. A distinctive characteristic of memory CD8^+^ T cells is that they rely on an oxidative metabolism and present a high SRC (19). Thus, we determined the OCR and ECAR of *in vitro*-generated Tc1 and Tc17 cells and found that Tc17 cells present a higher OCR and SRC than Tc1 cells (Figures 5A,B). Moreover, Tc17 cells exhibit higher levels of complex IV proteins compared to Tc1 cells (Figures 5C,D and Figure S2 in Supplementary Material), suggesting that they present an increased efficiency in respiration. All these data demonstrate that Tc17 cells rely mainly on an oxidative metabolism which has been already related to a memory CD8^+^ T cell phenotype.

## Discussion

Two main features characterize memory T cells; first, they are long-lived and maintained independently of stimulation, and second, they are functionally improved and respond more quickly and efficiently to repeated encounters with a specific pathogen than naive T cells. Also, depending on their migratory preferences, they can be classified into at least three subtypes: central memory T cells (T_CM_), effector memory T cells (T_EM_), and tissue-resident memory T cells (T_RM_) (20–22). Although the potential of naive CD8^+^ T cells to generate memory cells has been extensively studied, the potential of different CD8^+^ T cell effector subsets, especially Tc17 cells, to persist and become memory cells has not been fully characterized. Here, we show that Tc17 cells present functional features of T_CM_ cells such as the long-term maintenance, secondary expansion capacity, an oxidative metabolic profile, and a preferential trafficking within peripheral lymphoid tissues.

An essential property required for an efficient recall response is the ability of memory T cells to proliferate in response to antigenic stimulation (secondary expansion). In this work, we observed that unlike Tc1 cells, Tc17 lymphocytes were able to rapidly expand *in vivo* after secondary exposure to the antigen. This quality has been linked to the production and autocrine signaling of IL-2 (23), and consequently, it has been reported that T cells with a lower degree of differentiation such as T_CM_ produce higher levels of IL-2 in comparison to more differentiated T cells as T_EM_ (20, 24, 25). Moreover, it has been suggested that IL-2 production by T_CM_ cells is important in their enhanced proliferative capacity (26). Consistently, we observed that a high percentage of Tc17 lymphocytes generated *in vitro* produce IL-2, and even a fraction of the secondary progeny of Tc17 lymphocytes continues to produce IL-2 *in vivo* (12).

Recent studies have shown that CD25 (IL-2 receptor alpha chain) expression may determine the fate between memory versus effector cells (27, 28). It has been suggested that during the asymmetric distribution of CD25 during the first cell division of naive T cells, the population of CD25 low cells give rise to memory T lymphocytes (28). We have shown that Tc17 cells express low levels of CD25, which is consistent with the phenotype of memory precursors as previously reported by Arsenio and collaborators.

The ability of memory T lymphocytes to persist *in vivo* depends primarily on exposure to cytokines such as IL-7 and IL-15 (29), which promote cell survival by inducing the expression of anti-apoptotic proteins such as Bcl-2 (30). In this work we observed that Tc17 lymphocytes show a high expression of the IL-7 receptor alpha chain (CD127) *in vivo*, similar to that reported in the literature (11), suggesting that Tc17 lymphocytes may have advantages in long-term survival. In agreement with this idea, we observed that Tc17 lymphocytes expand more rapidly than Tc1 cells *in vitro* when cultured in the presence of IL-7 and also when transferred into Rag1-deficient mice. In support of this idea, the group of Klein has recently reported that vaccine-induced memory Tc17 cells express CD127, are durable and stable, and can persist for a year as functional IL-17A^+^ memory cells (31).

Interestingly, only Tc17 lymphocytes were found in lymphoid organs (spleen and lymph nodes) of mice after antigenic stimulation. This pattern of migration through the lymphoid organs is a hallmark of central memory T lymphocytes and depends on L-selectin (CD62L) and the chemokine receptor CCR7 expression (20, 21). Paradoxically, we observed that Tc17 lymphocytes generated *in vitro* do not express CD62L at the time of adoptive transfer (data not shown), nor the Klf-2 transcriptional factor, which commands CD62L expression. However, a fraction of the Tc17 lymphocytes (almost 30%) can re-express CD62L *in vivo* and maintain its expression even after secondary antigenic stimulation. In agreement, evidence from Arsenio and collaborators has shown that following antigenic stimulation, CD62L is initially down regulated and in time, it is subsequently upregulated in a population of memory T cells (28). This property of migrating through the lymphoid organs could allow the contact of the Tc17 lymphocytes with antigen-presenting cells and in this way favor the secondary response. In this line, previous studies have positively related the ability of the T lymphocytes to migrate to the lymph nodes with greater effectiveness in schemes of antitumor cellular immunotherapy (24, 32).

Evidence from the literature suggests that the Wnt-β-catenin pathway promotes the acquisition of the memory T cell phenotype while restricting the differentiation of CD8^+^ T lymphocytes into terminal effector T cells (32–34). Interestingly, in this work, we observed that Tc17 lymphocytes generated *in vitro* show a high expression of the transducers of the Wnt-β-catenin pathway, namely Tcf-7 and Lef-1, similar to the phenotype already reported in memory CD8^+^ T lymphocytes (32). This is especially relevant since studies conducted by the Restifo group have shown that the pharmacological activation of the Wnt pathway is sufficient to restrict terminal differentiation in CD8^+^ T lymphocytes, favoring the generation of highly undifferentiated memory T lymphocytes with a potent therapeutic potential (32, 35).

Bcl-6 is another important factor in maintaining the memory phenotype and has been reported to accumulate as CD8^+^ T lymphocytes transit from the effector into the memory phenotype (36). In agreement, overexpression of Bcl-6 in CD8^+^ T lymphocytes has shown to promote the generation of T_CM_ lymphocytes *in vivo* (37). The fact that Tc17 lymphocytes express higher levels of Bcl-6, Tcf-7, and Lef-1 could be a consequence of stimulation of the IL-6/STAT-3 pathway. Indeed, several cytokines related to the Tc17 program such as IL-6, IL-21, and IL-23 stimulate STAT-3 phosphorylation, which induces the expression of RORγt and IL-17, and represses the expression of granzyme B and IFN-γ (13, 14). Moreover, it has been reported that STAT-3 controls the expression of Bcl-6 (36) and Tcf-7 (38) in CD8^+^ and CD4^+^ T lymphocytes, respectively. In fact, STAT3-deficient CD8^+^ T lymphocytes are short-lived, fail to generate T_CM_ lymphocytes *in vivo*, and have a lower Bcl-6 expression compared to normal cells (36). Moreover, recent evidence from the group of Bosselut (39) has clearly demonstrated that STAT3 inhibits cytotoxic effector molecules such as granzymes and cytotoxic regulators such as T-bet and Eomes. Thus, signaling through the IL-6/STAT-3 pathway could contribute to the acquisition of the memory program observed in Tc17 lymphocytes.

Besides being involved in inhibiting Blimp-1 expression (40), Bcl-6 participates in the repression of genes that are critical to the glycolysis pathway, favoring the catalytic pathway (41). Memory CD8^+^ T cells are characterized by being highly dependent on a mitochondrial metabolism (19, 42), and the inhibition of glycolysis has been shown to improve the generation of memory T cells (43). It has been shown that memory CD8^+^ T cells present a high SRC, high OCRs, and mitochondrial mass compared to naive T cells, which allows explaining their long term survival and function (19, 42). The SRC constitutes the extra mitochondrial capacity available under stress conditions and increased work. In this line, our results demonstrate that Tc17 cells present higher OCR and SRC compared to Tc1 cells, thus suggesting that they exhibit a metabolic signature related to memory CD8^+^ T cells.

The results presented here demonstrate that following adoptive transfer, IL-17 production by Tc17 cells is lost when these cells enter effector tissues such as the peritoneal cavity; however, we detected a small fraction of Tc17 cells that still produce IL-17 in the spleen. Some reports from the literature demonstrate that IL-17 production by Tc17 and Th17 cells favors the recruitment of neutrophils into different tissues such as the lung in a murine model of lethal influenza infection (44), the tumor in a model of murine melanoma (10) and tumor draining lymph nodes in a murine colon carcinoma model (45). In all these reports, neutrophils have demonstrated to be pivotal in the induction of a protective immunity, thus IL-17 production by Tc17 cells in lymph nodes may be part of the effector mechanisms used by Tc17 cells to promote effective immune responses. Further studies are needed in order to demonstrate a role of IL-17 production by Tc17 cells in the recruitment of neutrophils to secondary lymph nodes.

Both phenotypic and functional properties observed in Tc17 lymphocytes (persistence, secondary expansion, lymph node migration, and self-renewal) strongly suggest that Tc17 lymphocytes possess qualities suitable for use in antitumor adoptive therapies. In agreement with this idea, our group together with other authors has shown that the adoptive therapy with Tc17 lymphocytes generated *in vitro* promotes the elimination of established melanoma tumors in mice (9, 10, 12) through an IFN-γ-dependent mechanism (10). Interestingly, following adoptive transfer, Tc17 cells gain the ability to produce IFN-γ. In light of these results, we can conclude that the role of Tc17 cells in this context may be to serve as a self-renewing reservoir of cells that will continuously give rise to Tc1-like cells. In spite of this, the therapeutic effectiveness of Tc17 lymphocytes has been questioned by studies showing that Tc1 lymphocytes are more efficient than Tc17 lymphocytes in the eradication of established tumors based on the number of transferred cells (10, 12). It is possible to reconcile these results by arguing that the high effector capacity of Tc1 lymphocytes is crucial to mediate a rapid elimination of tumor cells in a first stage, whereas the memory qualities of Tc17 lymphocytes would support a continuous attack in time, conferring immunity for longer periods.

## Ethics Statement

All mice were kept in the animal facility at Fundación Ciencia y Vida and maintained according to the “Guide to Care and Use of Experimental Animals, Canadian Council on Animal Care.” Animal work was carried out under institutional regulations of Fundacion Ciencia & Vida and Facultad de Ciencias, Universidad de Chile and was approved by the local ethics review committees.

## Author Contributions

FF-S designed the study, did experiments, analyzed the data, and wrote the manuscript; BC did experiments, analyzed the data, and wrote the manuscript; DF did experiments and analyzed the data; MVR, SN, and PC did experiments, analyzed the data, and wrote the manuscript; FG-C did experiments and analyzed the data; JC designed the study and analyzed the data; AL designed the study, analyzed the data, and wrote the manuscript; MR, MB, and DS designed the study, analyzed the data, and wrote the manuscript. All authors critically read the manuscript.

## Conflict of Interest Statement

The authors declare that the research was conducted in the absence of any commercial or financial relationships that could be construed as a potential conflict of interest.
