# Supplementary material for: In Vitro-Generated Tc17 Cells Present a Memory Phenotype and Serve As a Reservoir of Tc1 Cells In Vivo
Source: Front Immunol. 2018 Feb 8;9:209. doi: 10.3389/fimmu.2018.00209 (PMC5809442; doi:10.3389/fimmu.2018.00209)
Supplement: Supplementary file 1 [file Data_Sheet_1.docx]

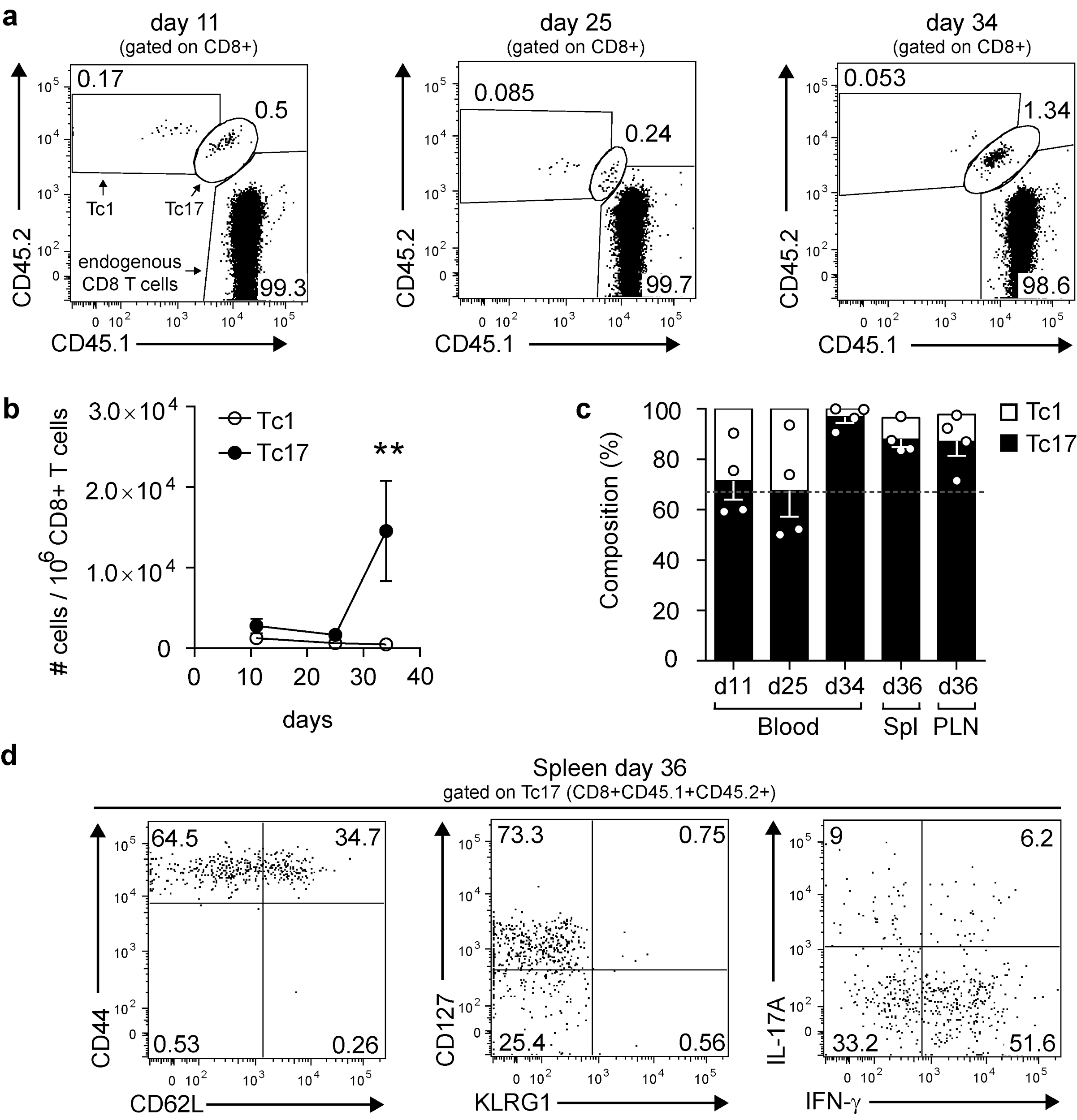
**Suplementary Figure 1. Tc17 cells expand rapidly following a secondary challenge.** Tc1 cells (CD45.2+) and Tc17 cells (CD45.1+/CD45.2+) were generated *in vitro* from OT-I mice. The cells were co-injected (2 x 10^6^ total cells) into CD45.1+ recipient mice. The mice were immunized i.p. with OVA protein at 1 and 30 days following adoptive transfer of Tc1 and Tc17 cells. (a) Dot plots depicting transferred Tc1 and Tc17 cells in blood at day 11, 25 and 34 after adoptive transfer. (b) Number of transferred Tc1 (white circles) and Tc17 cells (black circles) per 10^6^ CD8+ T cells at day 11, 25 and 34 in blood. (c) Percentage of transferred Tc1 (white bars) and Tc17 cells (black bars) at day 11, 25 and 34 in blood, and at day 36 in spleen and PLN. Two-way ANOVA and Bonferroni post-test, **p<0.01. n=4. (d) Cell surface markers and cytokine production was analyzed on transferred Tc17 cells obtained from spleen of recipient mice (representative from n=4).


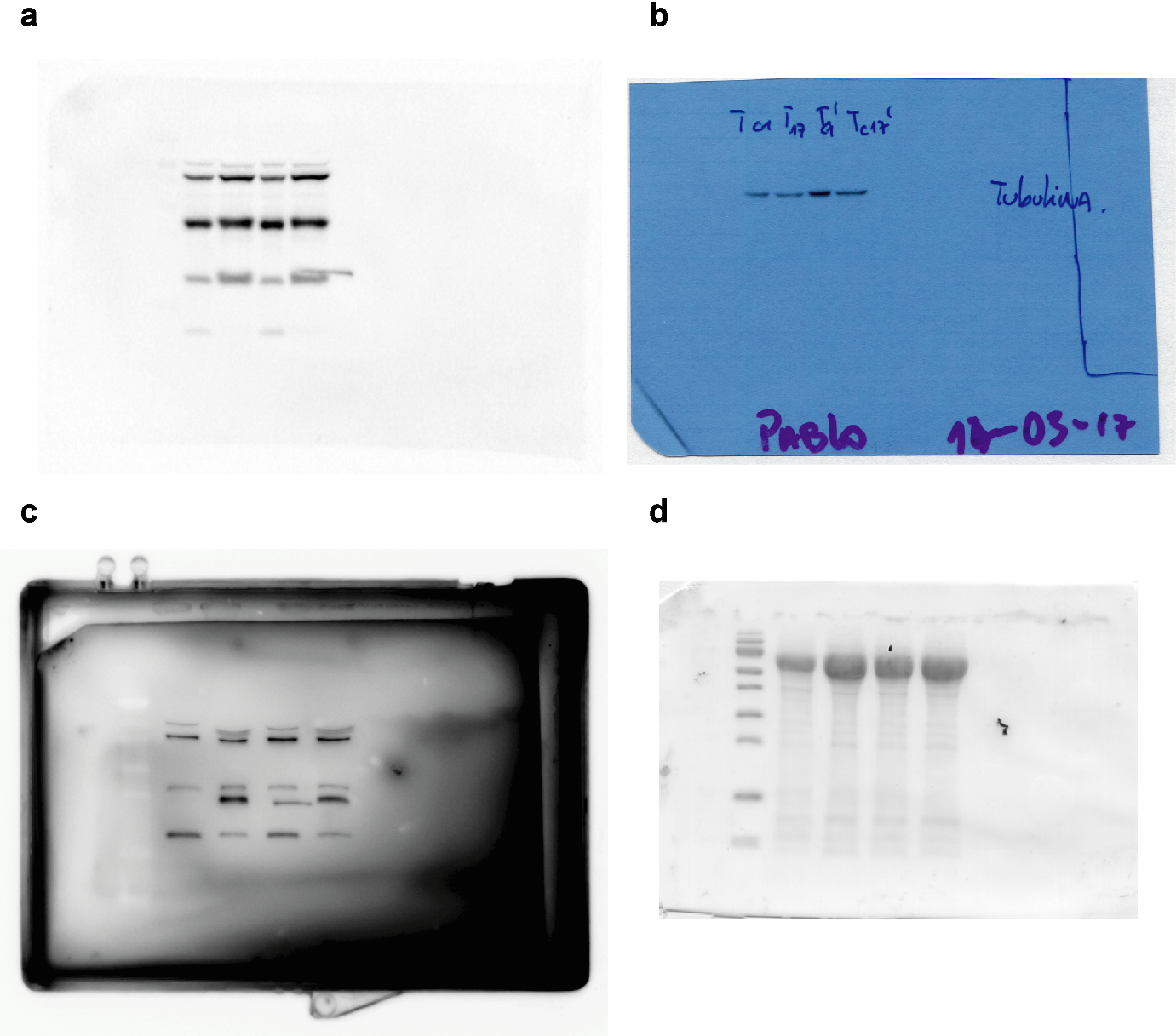
**Supplementary Figure 2. Original blots from figure 5**. (a) ETC Complex expression in Tc1 and Tc17 cells (n=1) in replicate. First and third lanes represent Tc1 cells, second and fourth lanes represent Tc17 cells. (b) Tubulin expression for blot presented in figure a. (c) ETC Complex expression in Tc1 and Tc17 cells (n=2). First and third lanes represent Tc1 cells, second and fourth lanes represent Tc17 cells. (d) Ponceau staining for blot presented in figure c.

References

Arsenio, J., Kakaradov, B., Metz, P.J., Kim, S.H., Yeo, G.W., and Chang, J.T. (2014). Early specification of CD8+ T lymphocyte fates during adaptive immunity revealed by single-cell gene-expression analyses. *Nat Immunol* 15(4)**,** 365-372. doi: 10.1038/ni.2842.

Brackett, C.M., Muhitch, J.B., Evans, S.S., Gollnick, S.O. (2013). IL-17 promotes neutrophil entry into tumor-draining lymph nodes following induction of sterile inflammation. *J Immunol* 191 (8), 4348-57. doi: 10.4049/jimmunol.1103621.

Cheuk, S., Wiken, M., Blomqvist, L., Nylen, S., Talme, T., Stahle, M., et al. (2014). Epidermal Th22 and Tc17 cells form a localized disease memory in clinically healed psoriasis. *J Immunol* 192(7)**,** 3111-3120. doi: 10.4049/jimmunol.1302313.

Crotty, S., Johnston, R.J., and Schoenberger, S.P. (2010). Effectors and memories: Bcl-6 and Blimp-1 in T and B lymphocyte differentiation. *Nat Immunol* 11(2)**,** 114-120. doi: 10.1038/ni.1837.

Cui, W., Liu, Y., Weinstein, J.S., Craft, J., and Kaech, S.M. (2011). An interleukin-21-interleukin-10-STAT3 pathway is critical for functional maturation of memory CD8+ T cells. *Immunity* 35(5)**,** 792-805. doi: 10.1016/j.immuni.2011.09.017.

Ciucci, T., Vacchio, M.S., Bosselut, R. (2017). A STAT3-dependent transcriptional circuitry inhibits cytotoxic gene expression in T cells. Proc Natl Acad Sci 114(50), 13236-13241. doi: 10.1073/pnas.1711160114.

Durant, L., Watford, W.T., Ramos, H.L., Laurence, A., Vahedi, G., Wei, L., et al. (2010). Diverse targets of the transcription factor STAT3 contribute to T cell pathogenicity and homeostasis. *Immunity* 32(5)**,** 605-615. doi: 10.1016/j.immuni.2010.05.003.

Feau, S., Arens, R., Togher, S., and Schoenberger, S.P. (2011). Autocrine IL-2 is required for secondary population expansion of CD8(+) memory T cells. *Nat Immunol* 12(9)**,** 908-913. doi: 10.1038/ni.2079.

Flores-Santibanez, F., Fernandez, D., Meza, D., Tejon, G., Vargas, L., Varela-Nallar, L., et al. (2015). CD73-mediated adenosine production promotes stem cell-like properties in mouse Tc17 cells. *Immunology* 146(4)**,** 582-594. doi: 10.1111/imm.12529.

Garcia-Hernandez Mde, L., Hamada, H., Reome, J.B., Misra, S.K., Tighe, M.P., and Dutton, R.W. (2010). Adoptive transfer of tumor-specific Tc17 effector T cells controls the growth of B16 melanoma in mice. *J Immunol* 184(8)**,** 4215-4227. doi: 10.4049/jimmunol.0902995.

Gattinoni, L., Lugli, E., Ji, Y., Pos, Z., Paulos, C.M., Quigley, M.F., et al. (2011). A human memory T cell subset with stem cell-like properties. *Nat Med* 17(10)**,** 1290-1297. doi: 10.1038/nm.2446.

Gattinoni, L., Zhong, X.S., Palmer, D.C., Ji, Y., Hinrichs, C.S., Yu, Z., et al. (2009). Wnt signaling arrests effector T cell differentiation and generates CD8+ memory stem cells. *Nat Med* 15(7)**,** 808-813. doi: 10.1038/nm.1982.

Goldrath, A.W., Sivakumar, P.V., Glaccum, M., Kennedy, M.K., Bevan, M.J., Benoist, C., et al. (2002). Cytokine Requirements for Acute and Basal Homeostatic Proliferation of Naive and Memory CD8+T Cells. *The Journal of Experimental Medicine* 195(12)**,** 1515-1522. doi: 10.1084/jem.20020033.

Hamada, H., Garcia-Hernandez Mde, L., Reome, J.B., Misra, S.K., Strutt, T.M., McKinstry, K.K., et al. (2009). Tc17, a unique subset of CD8 T cells that can protect against lethal Influenza challenge. J Immunol 182(6), 3469-81. doi: 10.4049/jimmunol.0801814.

Hamilton, S.E., and Jameson, S.C. (2012). CD8 T cell quiescence revisited. *Trends Immunol* 33(5)**,** 224-230. doi: 10.1016/j.it.2012.01.007.

Hinrichs, C.S., Kaiser, A., Paulos, C.M., Cassard, L., Sanchez-Perez, L., Heemskerk, B., et al. (2009). Type 17 CD8+ T cells display enhanced antitumor immunity. *Blood* 114(3)**,** 596-599. doi: 10.1182/blood-2009-02-203935.

Huber, M., Heink, S., Grothe, H., Guralnik, A., Reinhard, K., Elflein, K., et al. (2009). A Th17-like developmental process leads to CD8(+) Tc17 cells with reduced cytotoxic activity. *Eur J Immunol* 39(7)**,** 1716-1725. doi: 10.1002/eji.200939412.

Ichii, H., Sakamoto, A., Hatano, M., Okada, S., Toyama, H., Taki, S., et al. (2002). Role for Bcl-6 in the generation and maintenance of memory CD8+ T cells. *Nat Immunol* 3(6)**,** 558-563. doi: 10.1038/ni802.

Kalia, V., Sarkar, S., Subramaniam, S., Haining, W.N., Smith, K.A., and Ahmed, R. (2010). Prolonged interleukin-2Ralpha expression on virus-specific CD8+ T cells favors terminal-effector differentiation in vivo. *Immunity* 32(1)**,** 91-103. doi: 10.1016/j.immuni.2009.11.010.

Klebanoff, C.A., Gattinoni, L., Torabi-Parizi, P., Kerstann, K., Cardones, A.R., Finkelstein, S.E., et al. (2005). Central memory self/tumor-reactive CD8+ T cells confer superior antitumor immunity compared with effector memory T cells. *Proc Natl Acad Sci U S A* 102(27)**,** 9571-9576. doi: 10.1073/pnas.0503726102.

Kondo, T., Takata, H., Matsuki, F., and Takiguchi, M. (2009). Cutting edge: Phenotypic characterization and differentiation of human CD8+ T cells producing IL-17. *J Immunol* 182(4)**,** 1794-1798. doi: 10.4049/jimmunol.0801347.

Kryczek, I., Wei, S., Zou, L., Altuwaijri, S., Szeliga, W., Kolls, J., et al. (2007). Cutting Edge: Th17 and Regulatory T Cell Dynamics and the Regulation by IL-2 in the Tumor Microenvironment. *The Journal of Immunology* 178(11)**,** 6730-6733. doi: 10.4049/jimmunol.178.11.6730.

Kuang, D.M., Peng, C., Zhao, Q., Wu, Y., Zhu, L.Y., Wang, J., et al. (2010). Tumor-activated monocytes promote expansion of IL-17-producing CD8+ T cells in hepatocellular carcinoma patients. *J Immunol* 185(3)**,** 1544-1549. doi: 10.4049/jimmunol.0904094.

Liang, Y., Pan, H.F., and Ye, D.Q. (2015). Tc17 Cells in Immunity and Systemic Autoimmunity. *Int Rev Immunol* 34(4)**,** 318-331. doi: 10.3109/08830185.2014.954698.

Masopust, D., Vezys, V., Marzo, A.L., and Lefrancois, L. (2001). Preferential localization of effector memory cells in nonlymphoid tissue. *Science* 291(5512)**,** 2413-2417. doi: 10.1126/science.1058867.

Mueller, S.N., Gebhardt, T., Carbone, F.R., and Heath, W.R. (2013). Memory T cell subsets, migration patterns, and tissue residence. *Annu Rev Immunol* 31**,** 137-161. doi: 10.1146/annurev-immunol-032712-095954.

Muranski, P., Borman, Z.A., Kerkar, S.P., Klebanoff, C.A., Ji, Y., Sanchez-Perez, L., et al. (2011). Th17 cells are long lived and retain a stem cell-like molecular signature. *Immunity* 35(6)**,** 972-985. doi: 10.1016/j.immuni.2011.09.019.

Nanjappa, S.G., Heninger, E., Wuthrich, M., Gasper, D.J., and Klein, B.S. (2012). Tc17 cells mediate vaccine immunity against lethal fungal pneumonia in immune deficient hosts lacking CD4+ T cells. *PLoS Pathog* 8(7)**,** e1002771. doi: 10.1371/journal.ppat.1002771.

Nanjappa, S.G., McDermott, A.J., Fites, J.S., Galles, K., Wuthrich, M., Deepe, G.S., Jr., et al. (2017). Antifungal Tc17 cells are durable and stable, persisting as long-lasting vaccine memory without plasticity towards IFNgamma cells. *PLoS Pathog* 13(5)**,** e1006356. doi: 10.1371/journal.ppat.1006356.

Newell, E.W., Sigal, N., Bendall, S.C., Nolan, G.P., and Davis, M.M. (2012). Cytometry by time-of-flight shows combinatorial cytokine expression and virus-specific cell niches within a continuum of CD8+ T cell phenotypes. *Immunity* 36(1)**,** 142-152. doi: 10.1016/j.immuni.2012.01.002.

Oestreich, K.J., Read, K.A., Gilbertson, S.E., Hough, K.P., McDonald, P.W., Krishnamoorthy, V., et al. (2014). Bcl-6 directly represses the gene program of the glycolysis pathway. *Nat Immunol* 15(10)**,** 957-964. doi: 10.1038/ni.2985.

Sallusto, F., Lenig, D., Forster, R., Lipp, M., and Lanzavecchia, A. (1999). Two subsets of memory T lymphocytes with distinct homing potentials and effector functions. *Nature* 401(6754)**,** 708-712. doi: 10.1038/44385.

Srenathan, U., Steel, K., and Taams, L.S. (2016). IL-17+ CD8+ T cells: Differentiation, phenotype and role in inflammatory disease. *Immunol Lett* 178**,** 20-26. doi: 10.1016/j.imlet.2016.05.001.

Sukumar, M., Liu, J., Ji, Y., Subramanian, M., Crompton, J.G., Yu, Z., et al. (2013). Inhibiting glycolytic metabolism enhances CD8+ T cell memory and antitumor function. *J Clin Invest* 123(10)**,** 4479-4488. doi: 10.1172/JCI69589.

Tsai, J.P., Lee, M.H., Hsu, S.C., Chen, M.Y., Liu, S.J., Chang, J.T., et al. (2012). CD4+ T cells disarm or delete cytotoxic T lymphocytes under IL-17-polarizing conditions. *J Immunol* 189(4)**,** 1671-1679. doi: 10.4049/jimmunol.1103447.

van der Windt, G.J., Everts, B., Chang, C.H., Curtis, J.D., Freitas, T.C., Amiel, E., et al. (2012). Mitochondrial respiratory capacity is a critical regulator of CD8+ T cell memory development. *Immunity* 36(1)**,** 68-78. doi: 10.1016/j.immuni.2011.12.007.

van der Windt, G.J., O'Sullivan, D., Everts, B., Huang, S.C., Buck, M.D., Curtis, J.D., et al. (2013). CD8 memory T cells have a bioenergetic advantage that underlies their rapid recall ability. *Proc Natl Acad Sci U S A* 110(35)**,** 14336-14341. doi: 10.1073/pnas.1221740110.

Wherry, E.J., Teichgraber, V., Becker, T.C., Masopust, D., Kaech, S.M., Antia, R., et al. (2003). Lineage relationship and protective immunity of memory CD8 T cell subsets. *Nat Immunol* 4(3)**,** 225-234. doi: 10.1038/ni889.

Wu, P., Wu, D., Ni, C., Ye, J., Chen, W., Hu, G., et al. (2014). gammadeltaT17 cells promote the accumulation and expansion of myeloid-derived suppressor cells in human colorectal cancer. *Immunity* 40(5)**,** 785-800. doi: 10.1016/j.immuni.2014.03.013.

Yen, H.R., Harris, T.J., Wada, S., Grosso, J.F., Getnet, D., Goldberg, M.V., et al. (2009). Tc17 CD8 T cells: functional plasticity and subset diversity. *J Immunol* 183(11)**,** 7161-7168. doi: 10.4049/jimmunol.0900368.

Yu, Y., Cho, H.I., Wang, D., Kaosaard, K., Anasetti, C., Celis, E., et al. (2013). Adoptive transfer of Tc1 or Tc17 cells elicits antitumor immunity against established melanoma through distinct mechanisms. *J Immunol* 190(4)**,** 1873-1881. doi: 10.4049/jimmunol.1201989.

Zhang, Y., Hou, F., Liu, X., Ma, D., Zhang, Y., Kong, B., et al. (2014). Tc17 cells in patients with uterine cervical cancer. *PLoS One* 9(2)**,** e86812. doi: 10.1371/journal.pone.0086812.

Zhao, D.M., Yu, S., Zhou, X., Haring, J.S., Held, W., Badovinac, V.P., et al. (2010). Constitutive activation of Wnt signaling favors generation of memory CD8 T cells. *J Immunol* 184(3)**,** 1191-1199. doi: 10.4049/jimmunol.0901199.

Zhou, X., Yu, S., Zhao, D.M., Harty, J.T., Badovinac, V.P., and Xue, H.H. (2010). Differentiation and persistence of memory CD8(+) T cells depend on T cell factor 1. *Immunity* 33(2)**,** 229-240. doi: 10.1016/j.immuni.2010.08.002.

Zhuang, Y., Peng, L.S., Zhao, Y.L., Shi, Y., Mao, X.H., Chen, W., et al. (2012). CD8(+) T cells that produce interleukin-17 regulate myeloid-derived suppressor cells and are associated with survival time of patients with gastric cancer. *Gastroenterology* 143(4)**,** 951-962 e958. doi: 10.1053/j.gastro.2012.06.010.
